# Fast nanoDSF Tear Fluid Profiling: Toward Diagnosis of Age-Related Macular Degeneration

**DOI:** 10.3390/life16071048

**Published:** 2026-06-24

**Authors:** Philipp O. Tsvetkov, Veronika V. Tiulina, Elena N. Iomdina, Sergey Yu. Petrov, Nina Yu. Kushnarevich, Elena A. Suleiman, Olga M. Filippova, Oksana I. Markelova, Violetta N. Papyan, Timofey A. Chistyakov, Anton A. Bougaev, Natalia G. Shebardina, Mikhail L. Shishkin, Dmitriy V. Lipatov, Dmitry V. Chistyakov, Ivan I. Senin, Vladimir A. Mitkevich, Evgeni Yu. Zernii

**Affiliations:** 1 Inst Neurophysiopathol, INP, UMR 7051, Faculté des Sciences Médicales et Paramédicales, CNRS, Aix Marseille Univ, 13005 Marseille, France; 2 Belozersky Institute of Physico-Chemical Biology, Lomonosov Moscow State University, 1-40 Leninskye Gory, 119992 Moscow, Russia; 3 Helmholtz National Medical Research Center of Eye Diseases, 105062 Moscow, Russia; 4 Endocrinology Research Center, 117292 Moscow, Russia; 5 School of Nuclear Engineering, Purdue University, West Lafayette, IN 47907, USA; 6 Engelhardt Institute of Molecular Biology, Russian Academy of Sciences, 119991 Moscow, Russia

**Keywords:** biomarkers, diagnostics, tear fluid, nano-differential scanning fluorimetry, tear denaturation profiles, age-related macular degeneration, diabetic retinopathy, peripheral retinal dystrophy, dry eye syndrome

## Abstract

**Background**: Age-related macular degeneration (AMD) is the leading cause of irreversible vision loss in older adults. An important challenge is the recognition of its early asymptomatic stages and the monitoring of its progression, which requires reliable biomarkers. Growing evidence indicates that AMD-related biochemical changes are reflected in the proteome of tear fluid (TF). Although TF is a non-invasive and easily collectable diagnostic material, its proteomic analysis is complex and costly and therefore has limited clinical value. **Methods**: In this pilot single-center retrospective cross-sectional study, we developed a new method for dry AMD screening based on analysis of nano-differential scanning fluorimetry (nanoDSF) tear protein denaturation profiles (TDPs) within 15 min. The TDPs were recorded in representative groups of dry AMD patients (37% early, 48% intermediate, 15% geographic atrophy), and in control groups, including patients with refractive abnormalities (basic control), other retinal degenerative diseases (diabetic retinopathy, peripheral retinal dystrophy), or TF-affecting conditions (dry eye syndrome). High-dimensional TDP data were processed using unsupervised machine learning followed by k-means cluster analysis. **Results**: The presented pipeline distinguished AMD from the basic control with 74% accuracy and a sensitivity of 0.81 without relying on prior labels. The specificity of AMD detection was confirmed by its effective differentiation from diabetic retinopathy (72%; 0.74), peripheral retinal dystrophy (79%; 0.76) and dry eye disease (76%; 0.81). Classifying the AMD group from the entire population of other patients yielded an accuracy of 71% and a sensitivity of 85%, with a false-negative rate of only 15%. **Conclusions**: This study is a proof of concept for the nanoDSF-based approach, which can be considered a fast, cost-effective, and convenient tool for population screening for dry AMD, suitable for use in preventive medicine and public health.

## 1. Introduction

Age-related macular degeneration (AMD) is the leading cause of vision loss in older adults and represents a growing public health concern due to the aging population. The prevalence of the disease begins to rise after age 40, reaching 2% of the total population, and by age 75, one in four people is affected [1]. In Europe and North America, AMD is more common than in Asia [2]. Data on the gender distribution of the disease are conflicting, although most studies suggest that women are more likely to develop the condition [3,4]. Among the risk factors, the most notable is smoking: it is estimated that quitting smoking would reduce the incidence by approximately 1 million cases per year among both men and women [5]. AMD primarily damages the macula, impairing the ability to perform tasks that require sharp vision, such as reading, face recognition, and fine motor coordination, which leads to a loss of independence and a reduced quality of life. In about 10% of cases, the disease leads to complete vision loss, making it the third most common cause of irreversible blindness [1]. There are two main types of AMD. The dry or atrophic (non-vascular) form accounts for approximately 80–90% of cases; it progresses slowly, but almost lacks specific treatment except a few emerging therapies targeting the complement system [6]. The wet or exudative (vascular) form is less common and more often leads to severe vision loss due to choroidal neovascularization; however, in this case, there is an effective treatment based on intravitreal anti-vascular endothelial growth factor (VEGF) injections as well as photodynamic therapy and laser photocoagulation [7].

The pathophysiology of dry AMD involves drusen between Bruch’s membrane and the RPE, causing mechanical stress, disrupting phagocytosis, photoreceptor nutrition, and the retinoid cycle. This leads to lipofuscin accumulation in the RPE; its photoexcitation generates ROS, damaging lysosomal membranes and RPE integrity, ultimately killing photoreceptors and causing geographic atrophy [8]. About 3–10% of patients with large drusen develop neovascularization due to VEGF activation. Untreated wet AMD rapidly progresses to severe central vision loss from leakage, bleeding, and scarring [3,9,10]. It is important to note that at first presentation, patients commonly already have a severe form of the disease. For instance, in developing countries, nearly half of patients were shown to have advanced-stage AMD, and half of those with the wet form have discoid scarring, making them untreatable [3]. In addition, most patients with polypoidal choroidal vasculopathy are younger than 50 years of age, which raises concerns regarding early vision loss and the associated social and economic consequences [3]. These issues stem from the lack of population-based screening and fast, effective methods for the early diagnosis of AMD, as well as the inability to identify individuals at risk who require more frequent monitoring.

In most cases, the diagnosis of wet AMD is straightforward, based on the detection of signs of exudation (intraretinal and subretinal fluid, hemorrhages, fibrosis) using fundus biomicroscopy, fluorescein angiography, optical coherence tomography, and optical coherence tomography angiography. Large drusen, pigmentary changes, and geographic atrophy can also be detected using fundus biomicroscopy and color fundus photography. Fundus autofluorescence imaging, which allows for the detection of lipofuscin and other pathological fluorophores, is also useful for identifying areas of RPE atrophy [7]. At the same time, the early asymptomatic stages of AMD can be detected only in the presence of drusen and only using optical coherence tomography, which is unsuitable for rapid screenings. A growing body of evidence suggests that AMD is associated with systemic changes affecting the biochemical composition of plasma ([11]; for review, see [12,13]) and tear fluid (TF) [14,15,16], which has prompted a large-scale search for relevant biomarkers. The most accurate diagnosis can be achieved by simultaneously analyzing large patterns of such biomolecules (metallome, metabolome, lipidome, proteome) using modern omics approaches. However, the high-tech methods used for these purposes are complex, expensive, and out of reach in clinics.

Recently, we proposed a method for the rapid, non-invasive diagnosis of glaucoma based on the integrative analysis of the TF proteome using nano-differential scanning fluorimetry (nanoDSF), a label-free biophysical technique designed to measure the structural stability of proteins by tracking changes in intrinsic (tryptophan) fluorescence during their thermal denaturation [17]. NanoDSF analysis revealed characteristic two-peak tear denaturation profiles (TDPs), reflecting the complex process of thermal unfolding of the tear proteome components. In glaucoma, the shape and position of these peaks changed; therefore, clustering based on peak maximum values, combined with machine learning methods, enabled the detection of the disease in 70% of cases with a low number of false negatives (13.5%). Mechanistically, the changes in TDP were attributed to alterations in the composition of major tear proteins (primarily an increase in serum albumin levels, accompanied by a decrease in lysozyme C, lipocalin-1, and lactoferrin) as well as their low-molecular-weight ligands, including fatty acids and iron. Thus, TDP can be regarded as an integrative readout of proteomic, lipidomic, and metallomic alterations in TF, possessing diagnostic potential.

In this study we evaluated the feasibility of TDP analysis for rapid diagnosis of AMD, the retinal degenerative condition characterized by subtle changes in TF metallome and proteome [14,15,16]. The focus was on dry (atrophic, non-vascular) AMD, as this is typically the initial form of the disease and the target for early diagnosis. Our data indicate that TDP exhibits characteristic features in AMD that can be utilized for population-based screening in preventive medicine and public health.

## 2. Materials and Methods

### 2.1. Materials

The Schirmer tear strips were from Madhu Instruments (New Delhi, India). The NanoDSF quartz tubes were supplied by Biowe (Beijing, China).

### 2.2. Study Design and Participants

A single-center, retrospective, pilot cross-sectional study included 147 patients undergoing treatment at the Helmholtz National Research Center for Eye Diseases (Moscow, Russia). The cohort was recruited from September through December 2025. The AMD group included 27 patients, 37% of whom were diagnosed with early-stage AMD, 48% with intermediate-stage AMD, and 15% with geographic atrophy (late-stage AMD; Table 1). The exclusion criterion was the presence of diabetes mellitus. Patients with AMD did not receive any specific treatment, except for oral administration of special supplements containing vitamins and carotenoids, but underwent regular examinations (1–2 times a year) for monitoring using OCT. The control groups were as follows: 39 patients with refractive abnormalities (baseline control group), 29 with peripheral retinal dystrophy (PRD), 22 with dry eye syndrome (DES), and 30 with diabetic retinopathy (DR). The exclusion criterion for the basic control was a history of AMD or DES. Diagnosis was performed by qualified ophthalmologists (N.Y.K., E.A.S., O.M.F., O.I.M.). Refractive errors were detected using an autorefractometer (Nidek, Gamagori-shi, Aichi, Japan) under conditions of pharmacological mydriasis. PRD was diagnosed during fundus examination under mydriasis using a Goldman three-mirror lens, which allows visualization of the peripheral retinal segments. Examination of patients with AMD, PRD, and DR included ophthalmoscopy, biomicroscopy with color fundus photography (Eidon FA True Color Confocal Scanner centervue, CenterVue, Fremont, CA, USA), fundus autofluorescence imaging using confocal scanning laser ophthalmoscopy (color SLO, Mirante, Nidek, Gamagori-shi, Aichi, Japan), as well as optical coherence tomography (OCT; Spectralis OCT2, Heidelberg Engineering, Heidelberg, Germany). All procedures were conducted in accordance with the principles of the Declaration of Helsinki and the Association for Research in Vision and Ophthalmology (ARVO) guidelines for research involving human subjects, and were approved by the local ethics committee of the Helmholtz National Medical Research Center for Eye Diseases (protocol #68/1, 6 June 2024). Each participant signed a voluntary informed consent form.

### 2.3. TF Collection

TF was collected from patients using graduated Schirmer test strips, without anesthesia and without tear stimulation [17]. The procedure conditions were standardized for all participants: the same time of day (9–10 a.m.), fasting, the same medical staff, as well as identical lighting and air composition parameters. The strip was placed behind the lower conjunctival fornix for 5 min, after which the moistened fragment was cut off and +stored at −70 °C. To extract tear components, the strip was transferred to a 0.5 mL plastic microcentrifuge tube containing 30 μL of deionized water at a ratio of 1 μL of water per 1 mm of strip length. A hole was pre-punched in the bottom of the tube using a G30 needle and the tube was placed inside a 1.5 mL microcentrifuge tube and centrifuged at 14,000× *g* for 5 min at 4 °C. The resulting supernatant (TF sample) was used for further analysis.

### 2.4. Registration of TDPs

The tryptophan fluorescence intensities at 330 nm (F_330_) and 350 nm (F_350_) were measured as TF was heated from 35 to 95 °C. The final TDPs employed for analysis represented the temperature dependence of the first derivative of the F_350_/F_330_ ratio (∂(F_350_/F_330_)/∂T). TDPs were recorded using a PSA-16 (Biowe, Beijing, China) nanoDSF instrument. 20 μL of the sample was loaded into PSA-16 quartz tubes, and Heating was performed in the range from 35 °C to 95 °C at a rate of 0.3 K/s. The raw data obtained were exported into datasets containing the fluorescence intensities at wavelengths of 330 and 350 nm (F_330_ and F_350_), the ratio of these values (F_350_/F_330_), as well as their first derivatives with respect to temperature (∂F_330_/∂T, ∂F_350_/∂T, ∂(F_350_/F_330_)/∂T).

### 2.5. NanoDSF Data Analysis

TDPs were regularized (put on a common temperature grid), vectorized (flattened into fixed-length numerical vectors) and embedded into n dimensions by training an unsupervised autoencoder and using its n-unit bottleneck representation, wherein n varied from 2 to 22. The resulting n-dimensional embeddings were partitioned into two clusters using the k-means algorithm. For each pair of compared groups (basic control vs. AMD, age-adjusted basic control vs. AMD, PRD vs. AMD, DES vs. AMD, DR vs. AMD), the exported curves of the first derivative of the F_350_/F_330_ ratio (∂(F_350_/F_330_)/∂T) were interpolated onto a common temperature grid and converted into fixed-length numerical vectors containing 595 elements. Unsupervised dimensionality reduction was performed using an autoencoder implemented in Wolfram Mathematica 13.1 with the built-in DimensionReduce function and Method → “Autoencoder”; the latent dimensionality was systematically varied from 2 to 22. The autoencoder was used with default Wolfram Language settings, without manually defining a custom neural-network architecture or performing additional hyperparameter optimization. In the Wolfram Language implementation, the autoencoder is an encoder–decoder network that compresses input vectors into a low-dimensional latent representation and reconstructs the original input from this representation; the encoder and decoder are trained jointly by minimizing the discrepancy between the input and reconstructed data. The autoencoder-related settings, including network depth and maximum training rounds, were left at their default Automatic values. Random seeding was also left at the Wolfram Language default setting. All analyses were performed without supervised learning; class labels were not used at any stage.

## 3. Results

### 3.1. Patient Characteristics

**Figure 1:**
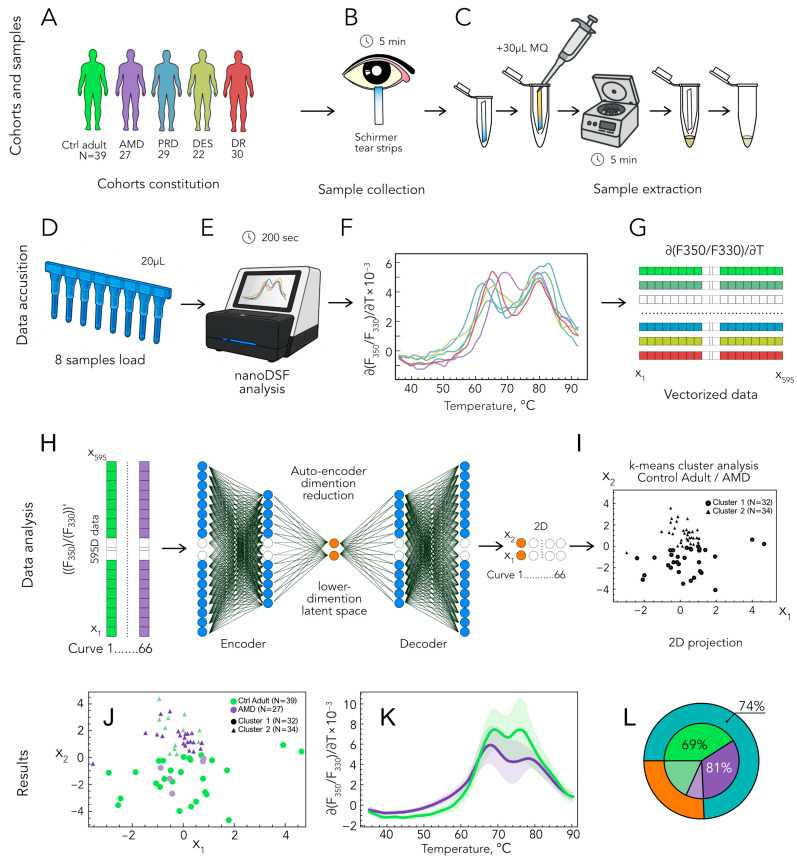
Algorithm for diagnosing AMD based on nanoDSF analysis of TF. TF samples are collected from patients (**A**) and control groups using Schirmer strips (**B**). The TF samples are extracted (**C**), placed in quarz tubes (**D**), and analyzed using nanoDSF instruments (**E**), yielding TDPs (**F**). TDPs were vectorized (**G**) and analyzed using unsupervised machine learning (autoencoder, (**H)**) and k-means clustering (**I**). (**J**–**L**) Classification of patients with AMD from basic control group. The resulting data clusters (**J**) and the corresponding distribution of TDPs (**K**) are shown. (**L**) Diagram illustrating the parameters distinguishing patients with AMD from the basic control group, including accuracy (outer ring) as well as sensitivity and specificity (inner sectors). Abbreviations: AMD, age-related macular degeneration; DES, dry eye syndrome; DR, diabetic retinopathy; PRD, peripheral retinal dystrophy; Ctrl, control; TF, tear fluid; TDPs, tear proteins denaturation profiles.

The AMD group included patients with characteristic signs of the disease, including pigmentary changes and drusen, with a mean age of 73.7 years (53–84 years) (Table 1, Figure 1A). The basic control group consisted of patients with refractive abnormalities, provided they had no history of AMD or DES. Since individuals with such characteristics are rare among the elderly and may have latent AMD, we recruited a control cohort spanning a wider age range (21–83 years; mean age 39 years). Age differences were accounted for in an additional analysis of subsets of patients with AMD (group “AMD (aged)”) and controls (group “Basic (aged)”), selected based on the criterion of being 60 years of age or older (Table 1). The other control groups included common concurrent retinal degenerative disorders, such as DR (30 patients, 32–85 years) and PRD (29 patients, 19–80 years). Although differential diagnosis between AMD and these conditions is generally not required, such discrimination may become beneficial in the early, clinically silent stages of retinal damage. Another control group consisted of patients with DES (22 patients, 20–62 years), the most common age-related ocular comorbidity that could bias the analysis results due to characteristic changes in TF composition. Finally, we used an expanded control group that included basic control individuals as well as patients with PRD and DES. The latter two conditions were included in the expanded control group based on their localized nature without systemic changes, which sets them apart from AMD and DR.

### 3.2. TF Profiling Using nanoDSF

TF samples were collected using a rapid method (5 min) that did not require any special preparation of the patient (Figure 1B). To ensure a reliable analysis of TF, we used the previously developed original procedure for its recovery, which preserves the composition of the tear proteins and their complexes. After the procedure was completed, the TF samples remained at the bottom of the microcentrifuge tube, from where they could be conveniently aspirated for loading into the nanoDSF instrument (Figure 1C–E) [17]. Among the data provided by the instrument, we next employed only curves representing the temperature dependence of the first derivative of the F_350_/F_330_ ratio (∂(F_350_/F_330_)/∂T) (referred to as TDPs). In all cases, the TDPs had two distinct peaks, but the positions of these peaks and the overall shape of the curves varied across the different groups (Figure 1F).

### 3.3. TDPs Classification Using Unsupervised Machine Learning

**Figure 2:**
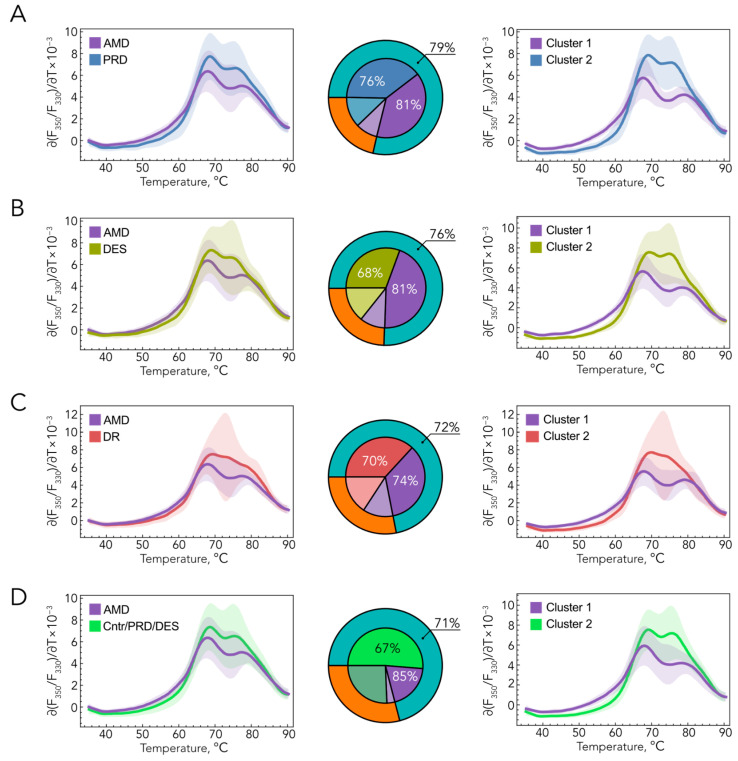
Classification of patients with AMD from control groups based on TF analysis using the nanoDSF. The results of processing of TDPs using unsupervised machine learning (**left**) and k-means clustering (**right**), along with diagrams (**center**) illustrating the parameters distinguishing AMD from PRD (**A**), DES (**B**), DR (**C**), and basic control/PRD/DES (**D**). Abbreviations: AMD, age-related macular degeneration; DES, dry eye syndrome; DR, diabetic retinopathy; PRD, peripheral retinal dystrophy; Ctrl, control.

To distinguish between AMD and control data, TDPs were processed using an unsupervised machine learning pipeline. An autoencoder reduced the data into low-dimensional embeddings, which were then partitioned into two clusters via k-means (Figure 1G,H). This approach effectively classified AMD patients from control individuals without using any prior labels. Post hoc comparison with clinical labels for the pair “basic control-AMD” showed that one cluster contained predominantly control TDPs (80%), whereas the other was enriched for AMD profiles (60%). This separation is encouraging; when applied as a diagnostic rule, majority-cluster assignment yielded 74% overall accuracy in our cohort (Figure 1I), with sensitivity 0.82, specificity 0.69, positive predictive value 0.65, negative predictive value 0.85, and an F1-score of 0.72 (Table 2). Importantly, when similar calculations were applied to the “basic control–AMD” pair, adjusted for age, only slightly lower identification accuracy was observed (62%; Table 2), while the proportion of false-negative results even decreased. These data confirm the overall reliability of distinguishing patients with AMD from ophthalmologically healthy individuals. The results of the analysis comparing the AMD group versus other control groups are presented in Figure 2. In all cases, the accuracy of the classification exceeded 72%, and the sensitivity exceeded 74% (Table 2). A comparison of AMD with a group comprising all other patients, excluding those with retinopathies (“Basic/PRD/DES”), showed an accuracy of 71% and a sensitivity of 85%, with a false-negative rate of only 15% (Table 2). The latter is particularly important in the context of screening for early-stage AMD, as our method makes it possible to reliably identify patients with the disease among healthy individuals and those with other common age-related eye conditions, so they can be referred for further testing (e.g., OCT).

## 4. Discussion

The rapid development of omics technologies in recent years has expanded diagnostic capabilities based on the use of biomarkers. The most promising and accurate approaches involve monitoring simultaneous changes in large sets of biomolecules with classification using machine learning techniques. The drawback of such methods is the high cost and complexity of omics research, and consequently, its inaccessibility to most clinics. Here, we propose a solution to this problem: an approach that enables the simultaneous examination of large sets of proteins/protein complexes by recording their integral denaturation profiles and classifying them using cluster analysis and machine learning methods. The approach is based on nanoDSF, a modern variant of differential scanning fluorimetry that allows for the rapid (within minutes) analysis of small sample volumes (10–20 μL). We have previously demonstrated the efficacy of an analogous method for diagnosing diseases associated with systemic biochemical changes, particularly cancer. For example, the analysis of plasma samples using nanoDSF, combined with AI-based data processing, successfully distinguished patients with glioma from healthy individuals [18]. Most recently, we demonstrated that a similar method can predict the development of breast cancer and could be used for multi-cancer differential diagnostics [19,20]. Furthermore, nanoDSF has been proposed as a universal platform for “liquid biopsy” diagnostics, in which systemic diseases (inflammatory, autoimmune, metabolic, etc.) can be recognized by characteristic changes in the thermal profiles of plasma or serum [19].

Notably, systemic changes in the proteome may be detected not only in blood but also in other biological fluids, particularly TF. Like plasma, TF reflects specific biochemical alterations in a wide variety of diseases, including cancers (e.g., breast cancer), autoimmune diseases, cardiovascular disease, type 2 diabetes mellitus, as well as neurological pathologies, such as Alzheimer’s disease [21,22]. Unsurprisingly, the composition of TF proteins also reflects changes associated with ophthalmic diseases, particularly those affecting the ocular surface (DES, Sjögren’s syndrome–associated dry eye, keratoconus, allergic conjunctivitis, infectious keratitis and other ocular surface infections) and the retina (glaucoma, diabetic retinopathy, AMD) [21,23,24]. Based on these findings, we recently adapted nanoDSF-based TF (TDP) analysis for the detection of glaucoma, one of the most common blindness-causing ophthalmic diseases affecting the optic nerve and retina [17]. Simple k-means clustering on TDPs identified 70% of primary open-angle glaucoma patients, while the introduction of machine-learning (AdaBoost) analysis reduced the number of false negatives to only 13.5%. Biochemical studies suggested that the high accuracy of glaucoma identification might rely on the sensitivity of the method not only to proteomic changes but also to the pattern of low-molecular-weight ligands of these proteins, such as metals and lipids [17]. Indeed, glaucoma is associated with significant changes in the metallomic, metabolomic, lipidomic, and proteomic composition of ocular tissues and fluids, including TF [25,26,27,28,29,30]. Similarly, AMD is characterized by changes in the metallome and proteome of TF, particularly metal-binding proteins (metallothionein-1A, S100A6, lactoferrin) (Fe, Mg, Cu) [14,16], and all these effects might have been captured by our TDP-based analysis. Importantly, the biochemical profile of AMD is unique, as it differed from all the controls used, including DES, which was expected to introduce the greatest bias in identification.

It should be noted that our pilot study has several limitations and can only be considered as proof of concept for the use of nanoDSF-based TF analysis as a tool for population-based screening for AMD. The first limitation relates to the age difference between patients with and without AMD, which may be an important confounding factor. Nevertheless, the difference between these patients reflects their actual distribution in clinical practice (the average age of onset for AMD is 70–80 years versus 40–60 years for DR/PDR), and the control groups may include patients with latent AMD. Furthermore, comparing the control group and the AMD group after adjusting for age allows for disease classification based on TDP analysis with a similar level of accuracy. The second limitation concerns the moderate size of the analyzed groups for obtaining reliable diagnostic conclusions, which underscores the need for further studies involving larger cohorts. Finally, our method yields slightly lower levels of accuracy (74%) and sensitivity (81%) than other established diagnostic approaches, such as structural OCT (sensitivity 92.4%) and color fundus photography/fundus angiography (accuracy 83.7–88.5%; sensitivity 83.7–88.5%) or blood testing for a panel of metabolites (accuracy 87.5%) [31,32,33].

Nevertheless, the proposed approach offers a number of practical advantages that are important for implementation in the field of preventive medicine and public health. Thus, it does not require costly clinical (OCT; color fundus photography/fundus angiography) or biochemical (mass spectrometry) examination and, unlike blood collection, TF sampling is a completely non-invasive procedure that does not require special clinical facilities, consumables, or qualified personnel. Moreover, it is convenient to perform this procedure exactly in an ophthalmology department. A standard ophthalmic examination of elderly patients typically involves the Schirmer test, which is used to diagnose DES [34]. Instead of discarding the moistened test strip, we suggest using it to extract TF for subsequent analysis of TDPs. This will allow patients to be screened for suspected glaucoma [17] and AMD (this study) without additional effort or discomfort on their part, and in the case of a positive result, to classify them into the appropriate risk groups with recommendations for further, more detailed, and costly examinations.

## Figures and Tables

**Table 1:** Characteristics of experimental groups.

Parameter		AMD	AMD (Aged)	Controls				
				Basic (Total)	Basic (Aged)	DR	PRD	DES
Participants		27	24	39	23	30	29	22
Age, years		73.7 ± 10.9	76.9 ± 5.5	53.7 ± 16.8	69.3 ± 8.1	56.2 ± 16.7	40.5 ± 16.3	46.4 ± 13.7
Gender, %	Men	33.3	29.2	33.3	35.0	20	37.9	54.5
	Women	66.7	70.8	66.7	65.0	80	62.1	45.5

Abbreviations: AMD, age-related macular degeneration; DES, dry eye syndrome; DR, diabetic retinopathy; PRD, peripheral retinal dystrophy.

**Table 2:** Classification of AMD and control patients based on TDP analysis.

Control Groups	Patients’ Distribution					Accuracy (95% CI)	Sensitivity (95% CI)	Specificity (95% CI)	F1-Score (95% CI)
	Total	TN	FN	FP	TP				
Basic control	66	27	5	12	22	0.74 (0.63–0.83)	0.81 (0.63–0.92)	0.69 (0.54–0.81)	0.72 (0.58–0.84)
Basic control aged	47	12	7	11	17	0.62 (0.47–0.74)	0.71 (0.51–0.85)	0.52 (0.33–0.71)	0.65 (0.49–0.79)
DES	49	15	5	7	22	0.76 (0.62–0.85)	0.81 (0.63–0.92)	0.68 (0.47–0.84)	0.79 (0.65–0.89)
PRD	56	22	5	7	22	0.79 (0.66–0.87)	0.76 (0.58–0.88)	0.82 (0.63–0.92)	0.79 (0.65–0.89)
DR	57	21	7	9	20	0.72 (0.59–0.82)	0.74 (0.55–0.87)	0.70 (0.52–0.83)	0.71 (0.56–0.84)
Basic/PRD/ DES	117	60	4	30	23	0.71 (0.62–0.78)	0.85 (0.68–0.94)	0.67 (0.56–0.76)	0.58 (0.43–0.70)

Abbreviations: AMD, age-related macular degeneration; TDP, tear denaturation profile; DES, dry eye syndrome; DR, diabetic retinopathy; PRD, peripheral retinal dystrophy; TN, true negative; FN, false negative; FP, false positive; TP, true positive.

## Data Availability

The de-identified nanoDSF curve data and the Mathematica workflow used for the analysis are available from the corresponding authors upon reasonable request, subject to institutional and ethical restrictions related to patient-derived data.

## Abbreviations

The following abbreviations are used in this manuscript:

AMD	age-related macular degeneration
DES	dry eye syndrome
DR	diabetic retinopathy
nanoDSF	nano-differential scanning fluorimetry
OCT	optical coherence tomography
PRD	peripheral retinal dystrophy
RPE	retinal pigment epithelium
ROS	reactive oxygen species
TDPs	tear denaturation profiles
TF	tear fluid
VEGF	vascular endothelial growth factor
